# Rhizosphere microbial ecological characteristics of strawberry root rot

**DOI:** 10.3389/fmicb.2023.1286740

**Published:** 2023-11-16

**Authors:** Meilin Zhang, Zirong Kong, Huijing Fu, Xiaolong Shu, Quanhong Xue, Hangxian Lai, Qiao Guo

**Affiliations:** College of Natural Resources and Environment, Northwest A&F University, Yangling, China

**Keywords:** *Fragaria* × *ananassa* Duch., root rot, culturable microorganisms, high-throughput sequencing, microbial network

## Abstract

**Introduction:**

Strawberry (*Fragaria* × ananassa Duch.) holds a preeminent position among small fruits globally due to its delectable fruits and significant economic value. However, strawberry cultivation is hampered by various plant diseases, hindering the sustainable development of the strawberry industry. The occurrence of plant diseases is closely linked to imbalance in rhizosphere microbial community structure.

**Methods:**

In the present study, a systematic analysis of the differences and correlations among non-culturable microorganisms, cultivable microbial communities, and soil nutrients in rhizosphere soil, root surface soil, and non-rhizosphere soil of healthy and diseased strawberry plants affected by root rot was conducted. The goal was to explore the relationship between strawberry root rot occurrence and rhizosphere microbial community structure.

**Results:**

According to the results, strawberry root rot altered microbial community diversity, influenced fungal community composition in strawberry roots, reduced microbial interaction network stability, and enriched more endophytic-phytopathogenic bacteria and saprophytic bacteria. In addition, the number of bacteria isolated from the root surface soil of diseased plants was significantly higher than that of healthy plants.

**Discussion:**

In summary, the diseased strawberry plants changed microbial community diversity, fungal species composition, and enriched functional microorganisms significantly, in addition to reshaping the microbial co-occurrence network. The results provide a theoretical basis for revealing the microecological mechanism of strawberry root rot and the ecological prevention and control of strawberry root rot from a microbial ecology perspective.

## 1. Introduction

Strawberry (*Fragaria* × *ananassa* Duch.), a herbaceous plant belonging to the family Rosaceae and the genus *Fragaria*, has gained prominence globally as a cultivated fruit due to its short growth cycle, delightful taste, substantial consumer demand, and high economic returns. In fact, strawberries are one of the most extensively cultivated fruit crops globally (Whitaker et al., 2020). As the strawberry industry advances, the cultivation area of strawberry has expanded, accompanied by prolonged planting durations. Consequently, challenges associated with strawberry crop rotation have intensified, leading to the prevalence of various diseases.

Strawberry root rot is one of the major diseases encountered in strawberry production. Strawberry root rot is caused by *Phytophthora fragariae* and various soil-borne pathogens, including *Fusarium oxysporum* and *Rhizoctonia solani* (Fang et al., 2013; Juber et al., 2014; Chen et al., 2017b). When the disease is severe, the entire strawberry garden is infected and the harvest is destroyed. Strawberry root rot disease occurs rapidly, and the symptoms of the diseased plants are not obvious in the early stages. In the middle and late stages of the disease, the plants wither and wilt quickly, especially after rain (Sánchez et al., 2013; Dinler et al., 2016). Preventative measures constitute the primary approach of managing strawberry root rot. The etiology behind the malady is multifaceted, encompassing factors such as inadequate seedbed drainage, soil compaction and salinization resulting from excessive fertilization, the accumulation of harmful substances due to successive planting, and disruption of soil microbial communities. Such factors collectively decrease plant resilience, in turn triggering root rot occurrence (Osman and Osman, 2013; Shankar et al., 2014; Abbas et al., 2022).

Soil has an intimately intertwined relationship with plant health, with its physical, chemical, and biological properties directly influencing plant growth and development (Timmis and Ramos, 2021). Within such a context, soil microorganisms have a pivotal role as decomposers within the soil ecosystem. Soil microbes play vital roles in plant growth, development, and stress resistance (Sun et al., 2017). Philippot et al. (2013) defined the rhizosphere as an ecological system comprised of the interactions among plant roots, rhizosphere soil, and rhizosphere microbes. This triad forms a dynamically balanced ecosystem that influences plant growth and health profoundly. Monocropping disrupts the equilibrium through plant–soil negative feedback, altering the soil environment and fostering plant disease (van der Putten et al., 2013). Imbalance and reduced adaptability within the plant-root-microbiota-soil system underlies the continuous cropping obstacle phenomenon (Cook and Baker, 1983; Weller et al., 2002). Consequently, soil microbial community structure represents a key indicator of soil health (Nannipieri et al., 2003).

Beneficial rhizosphere microbes establish symbiotic relationships with plants in the roots. They participate in functions such as nutrient absorption, immune defense, disease resistance, and growth regulation in plants (Bulgarelli et al., 2013). Harmful microorganisms, conversely, such as soil-borne plant pathogens, can adversely affect crops (Pieterse et al., 2014), by causing root infections and subsequently diminishing crop yield and quality. Hence, understanding the microbial ecological mechanisms underlying their occurrence is essential for effective biological control of strawberry root diseases. Current research on plant disease management mainly focuses on the suppressive effects of chemical and biological control methods on pathogens (Anandhakumar and Zeller, 2008; Parikka et al., 2017); however, investigations on the mechanistic aspects of strawberry disease from healthy and diseased root ecosystem perspectives are scarce. Therefore, studying root ecosystem dynamics in the context of strawberry continuous cropping and soil-borne disease carries significant implications for biocontrol strategies.

The present study investigated differences in soil nutrients and non-culturable and culturable microbial communities in rhizosphere soil and root surface soil between healthy and diseased strawberries. The aim was to shed light on the microbial ecological mechanisms behind strawberry root rot occurrence and provide insights that could facilitate their biocontrol. Furthermore, the bacteria isolated and identified from the root surface and rhizosphere soil could provide valuable resources for biocontrol and pave the way for further research and development.

## 2. Materials and methods

### 2.1. Soil sample collection and preparation

The soil samples from both diseased and healthy strawberry plants were collected at the Strawberry Experimental Base in Huyi District, Xi’an City, Shaanxi Province, China (latitude 34°6′5″ N, longitude 108°31′49″ E, elevation 430 m, and an average annual temperature of 14.3°C). The strawberry variety used in the present study was “Hongyan.” The progression of strawberry disease is illustrated in Supplementary Figure 1. Field observations indicated that the roots of diseased plants turned black, with reduced lateral root numbers. Upon dissection, the central column of the infected plants exhibited a distinct reddish-brown color, whereas in healthy plants, the central column appeared yellowish-white (Supplementary Figure 2). Diseased plants were selected based on clear strawberry root rot symptoms, including wilting of the aboveground parts and reddening of the core in the underground roots. Healthy plants were selected around the diseased ones (within <1 m) and were characterized by robust growth. There were six replicates for the diseased and healthy plants.

The strawberry root system was shaken to shake off the soil not closely attached to the root system, and the rhizosphere soil closely attached to the root system was collected as the rhizosphere soil. As for the root surface soil, visible soil was removed, and the roots were suspended in sterile distilled water. The mixture was subjected to ultrasonic cleaning for 10 min (160 W, 30/30s). The resulting soil solution was centrifuged at 12,000 g for 10 min to remove the supernatant, leaving behind the tightly adhered root surface soil (Edwards et al., 2015; Khan et al., 2019). Non-rhizosphere soil was collected randomly from six points within the 5–20 cm plow layer that was not influenced by root presence. Rhizosphere, root surface, and non-rhizosphere soil samples were stored at 4°C and −80°C for subsequent soil microbial analyses. Additionally, some soil samples were air-dried, ground, and sieved for use in soil nutrient and enzyme activity analyses. Hereafter, the rhizosphere soil of healthy plants is referred to as “HR,” the root surface soil of healthy plants as “HS,” the rhizosphere soil of diseased plants as “DR,” the root surface soil of diseased plants as “DS,” and the non-rhizosphere soil as “NR.”

### 2.2. Soil physicochemical property analysis

Following the collection described in section 2.1, the HR, DR and NR soil sample were air-dried and ground, sieved through a 2-mm and 0.25-mm sieve. The meticulously prepared soil samples were then used for the determination of soil physicochemical indicators. Organic matter content was determined using the potassium dichromate volumetric method (Inclán et al., 2007). Total nitrogen content was determined using the Kjeldahl method. Similarly, total phosphorus content was assessed using the HClO_4_-H_2_SO_4_ digestion method followed by molybdenum antimony colorimetry. Total potassium content was determined using the NaOH fusion-flame atomic absorption spectrometry technique. Alkali hydrolyzable nitrogen was quantified using the diffusion method (Restrepo et al., 2016). The evaluation of rapidly available phosphorus was accomplished using the sulfuric acid-molybdenum antimony colorimetric procedure. Available potassium content was assessed using flame photometry. Finally, the pH of the soil samples was measured using the potentiometric method (Read et al., 2003; Shrestha and Lal, 2011).

### 2.3. Determination of culturable soil microbial abundance

The quantification of cultivable bacterial populations in the soil was achieved using the plate dilution spread method. Bacteria were cultivated on LB agar medium at 30°C for 72 h. Subsequently, bacterial colony-forming units (CFUs) that developed on the counting plates were converted into CFUs per gram of dry soil (CFU/g) to represent microbial abundance. After enumeration, individual bacterial colonies were carefully selected from the bacterial plates cultured from the rhizosphere, root surface soil, and non-rhizosphere soil samples of both diseased and healthy plants. The selected colonies were subjected to purification and isolation, followed by thorough identification.

Bacterial Molecular Identification: Bacteria were inoculated into liquid LB medium and placed in a constant temperature oscillating incubator (30°C, 150 rpm) for 48 h until the culture medium became turbid. DNA was extracted using the freeze–thaw method to obtain DNA templates (Cullen and Hirsch, 1998). After extracting bacterial DNA and conducting quality assessment using NanoDrop 2000 (Thermo Fisher Scientific, Wilmington, DE, USA), the extracted bacterial genomes were used as templates for PCR amplification using specific primers 27F/1492R targeting the bacterial 16S rDNA gene (Lane, 1991). Amplification reactions (25-μL volume) consisted of 2 × T5 Super PCR Mix (Basic) 12.5 μL, forward primer (10 μM) 1 μL, reverse primer (10 μM) 1 μL, template DNA (100 ng)1 μL and DNase-free water 9.5 μL. The PCR amplification was performed as follows: initial denaturation at 94°C for 3 min, followed by 30 cycles of denaturing at 95°C for 30 s, annealing at 58°C for 30 s and extension at 72°Cfor 45 s, and single extension at 72°C for 10 min, and end at 4°C. The obtained 16S rDNA sequences of the bacteria were aligned with the NCBI nucleotide database through online BLAST comparison (Zhang et al., 2010; BLAST: Basic Local Alignment Search Tool^1^), providing insights into the genetic relationships among different bacterial strains.

### 2.4. Soil DNA extraction and third-generation long amplicon sequencing

Each of the 24 samples, including “HS,” “DS,” “HR,” and “DR” had 6 replicates each were stored at −80°C. Microbial community DNA was extracted according to the instructions of the E.Z.N.A.® Soil DNA kit (Omega Bio-tek, Norcross, GA, United States). The extracted DNA served as the template for the amplification of both the full-length 16S rRNA gene and the full-length ITS region through PCR. After PCR purification, the purified products were quantified using a Quantus™ Fluorometer (Promega, United States). Subsequently, adhering to the sequencing requirements of each sample, the products were mixed at corresponding proportions. Library preparation was conducted using the SMRTbell® Express Template Prep Kit 2.0 (PacBio, Menlo Park, CA, United States), involving steps such as (1) DNA damage repair, (2) end repair, and (3) adapter ligation. Sequencing was performed using the Pacbio Sequel II System (Shanghai Meiji Biopharmaceutical Technology Co., Ltd.). The resulting PacBio raw data were subreads, and the Circular Consensus Sequence (CCS) sequences were obtained using the SMRTLink 8.0 (PacBio) with parameters set as minfullpass =3 and minPredictedAccuracy = 0.99. To distinguish data from different samples, barcode sequences were employed, followed by length filtering to retain sequences ranging from 1,000 to 1,800 bp for bacteria and 300 to 900 bp for fungi.

### 2.5. Data processing

The soil physicochemical properties and counts of cultivable microorganisms indicators were analyzed for significant differences among different soil samples using IBM SPSS Statistics 25 (IBM Corp., Armonk, NY, United States) using the Duncan’s test (*p* < 0.05). Analysis of high-throughput sequencing data was conducted on the Majorbio Cloud Platform^2^ using the following comprehensive procedures:

Sequence quality control: Using UPARSE 7.1 (http://drive5.com/uparse/version 7.1; Stackebrandt and Goebel, 1994; Edgar, 2013), sequences were clustered into Operational Taxonomic Units (OTUs) based on a 97% similarity threshold, with meticulous removal of chimeric sequences. Moreover, the sequences annotated as chloroplasts and mitochondria across all samples were systematically excluded, followed by subsampling to ensure consistency in sample sequence numbers.

Species annotation: Using the Silva138.1/16s_bacteria^3^ (Dueholm et al., 2020) and unite8.0/its_fungi (Release 8.0 http://unite.ut.ee/index.php; Nilsson et al., 2019). the sequences contained in each sample were meticulously aligned to determine the diversity of species present, accomplished thorough species annotation.

Diversity analysis: Leveraging the MOTHUR software,^4^ a comprehensive suite of alpha diversity indices was computed for each sample, including Shannon Wiener, Chao1, and Gini-Simpson indices. For beta diversity analysis, the Bray-Curtis distance-based Principal Coordinates Analysis (PCoA) was performed, yielding graphical representations.

Microbial correlation network construction: Through amalgamation of bacterial and fungal species annotation data tables, a curated set of species that exhibited presence in over 50% of all samples was retained. The data were subjected to comprehensive microbial correlation network analysis at the genus level, using the Hmisc package in R-4.3.1 (R Foundation for Statistical Computing, Vienna, Austria; Chen et al., 2017a). The evaluation entailed computation of Spearman correlation coefficients, thereby generating matrices containing correlation values (r) and significance levels (p). Further refinement involved the inclusion of species data that satisfied criteria with |r| > 0.90 and *p* < 0.05, followed by importing the data into the Gephi software (version 0.10.1) for the construction of correlation network graphs and the calculation of their topological properties.

Network natural connectivity assessment: Assessment of network natural connectivity was performed using fastnc^5^ (Peng and Wu, 2016; Wu et al., 2021). This involved rigorously testing the network’s resilience by randomly eliminating nodes within the network, ranging from 1% to 99%, through an iterative simulation process conducted 1,000 times. The resulting averaged values were then utilized for the calculation and evaluation of the network’s natural connectivity and overall stability.

Functional prediction: The FAPROTAX database was used for comprehensive 16S functional analysis to predict functional attributes (Sansupa et al., 2021). Additionally, the FUNGuild database was harnessed for the systematic prediction of ITS functional attributes (Nguyen et al., 2016).

## 3. Results

### 3.1. Soil physicochemical properties

The various physicochemical properties of strawberry root rot-diseased plant rhizosphere soil, healthy plant rhizosphere soil, and non-rhizosphere soil are presented in Table 1. As shown in the table, significant differences were observed in TN (total nitrogen) and AN (available nitrogen) between HR and DR (*p* < 0.05), while other physicochemical indicators showed minimal differences and were not statistically significant. HR and DR exhibited higher TN, SOM (Soil Organic Matter), and OP (Olsen Phosphorus) values than NR. The pH of HR and DR was significantly higher than that of NR (*p* < 0.05). However, AK (available potassium) content was higher in NR than in both HR and DR.

**Table 1:** Physical and chemical properties of rhizosphere soil of healthy plants, rhizosphere soil of diseased plants, and non-rhizosphere soil of strawberry.

Treatment	HR	DR	NR
TN (g/kg)	1.06 ± 0.08a	1.02 ± 0.04ab	0.96 ± 0.01c
SOM (g/kg)	19.15 ± 2.15a	19.07 ± 0.85a	17.50 ± 0.34a
AN (mg/kg)	72.93 ± 4.75a	68.99 ± 4.26a	72.93 ± 3.51a
OP (mg/kg)	26.58 ± 2.66a	26.63 ± 7.91a	20.96 ± 1.46a
AK (mg/kg)	123.83 ± 11.88a	123.83 ± 7.20a	131.17 ± 5.61a
pH	6.59 ± 0.21a	6.54 ± 0.20a	6.25 ± 0.04b

Values followed by different letters within the columns are significantly different according to Duncan’s Test (*p* < 0.05), HR, DR and NR are healthy rhizosphere soil, diseased rhizosphere soil, and non-rhizosphere soil, respectively.

### 3.2. Soil microbial diversity

Using high-throughput sequencing technology, a total of 1,348,655 fungal sequences with 849,575,807 bases and an average sequence length of 629 bp, as well as 890,840 optimized bacterial sequences with 1,286,151,991 bases and an average sequence length of 1,443 bp, were obtained from 24 samples of healthy strawberry rhizosphere soil, healthy plant root surface soil, diseased plant rhizosphere soil, and diseased plant root surface soil. A total of 19,851 fungal OTUs and 4,728 bacterial OTUs were generated from the 24 soil samples.

**Figure 1:**
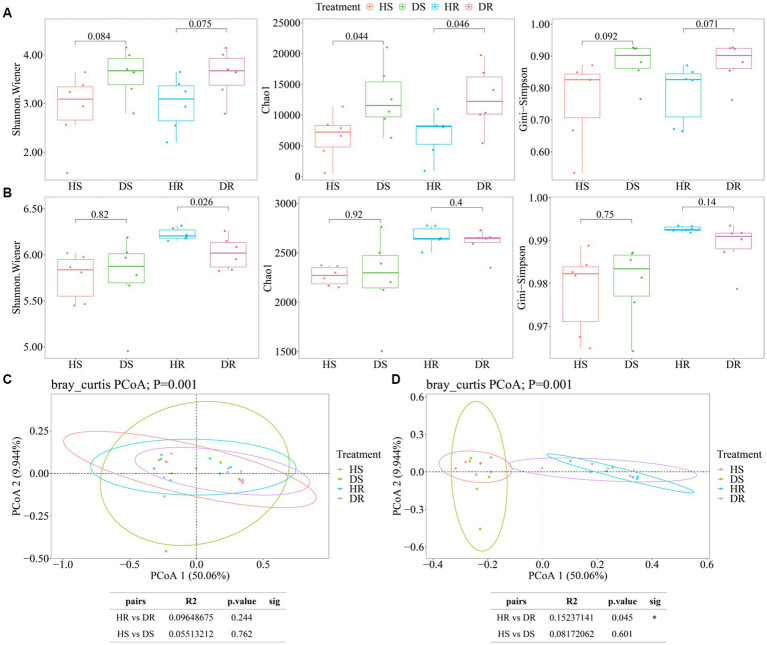
Microbial alpha diversity. The α-diversity indices of soil fungal **(A)** and bacterial **(B)** communities in the root surface soil and rhizosphere soil of healthy and diseased strawberry plants. **(C,D)** Comparison of beta diversity related to root surface soil and rhizosphere soil between healthy and diseased strawberry plants, **(C)** fungal beta diversity; **(D)** Bacterial beta diversity. Alpha diversity: The horizontal bars within boxes represent medians. The tops and bottoms of boxes represent the 75th percentiles and 25th percentiles, respectively. The asterisk above the horizontal line represents a significant difference between the two groups, ^*^p < 0.05, ^**^p < 0.01, and ^***^p < 0.001. Beta diversity: Different color points represent the samples in each group, and the circle in the figure is a confidence ellipse at a confidence level of 95%.

Soil microbial community diversity in different ecological niches of healthy strawberry plants and those affected by root rot are illustrated in Figure 1. The results reveal diversity variations in fungal and bacterial communities among different soil samples. The alpha diversity of fungal communities in both Ds and DR was higher than those in HS and HR; however, there were no significant differences in alpha diversity among different ecological niches (root surface soil and rhizosphere soil) for either healthy or diseased plants (Figure 1A). The alpha diversity of bacterial communities exhibited distinct trends when compared with those in fungal communities. In the root surface soil, the alpha diversity of bacterial communities showed no significant difference between healthy and diseased plants. However, in rhizosphere soil, the alpha diversity of HR bacterial communities was significantly higher than that of DR bacterial communities (*p* < 0.05; Figure 1B).

Principal coordinates analysis (PCoA) results based on Bray-Curtis distance are illustrated in Figure 1C for fungal communities, revealing no significant differences among soil samples. In contrast, for bacterial communities (Figure 1D), there were no significant differences in beta diversity among bacterial colonies in root surface soil between healthy and diseased plants (*p* > 0.05). However, significant differences were observed in bacterial community beta diversity between healthy and diseased plants in rhizosphere soil (*p* = 0.041, Adonis test).

### 3.3. Soil microbial species composition

**Figure 2:**
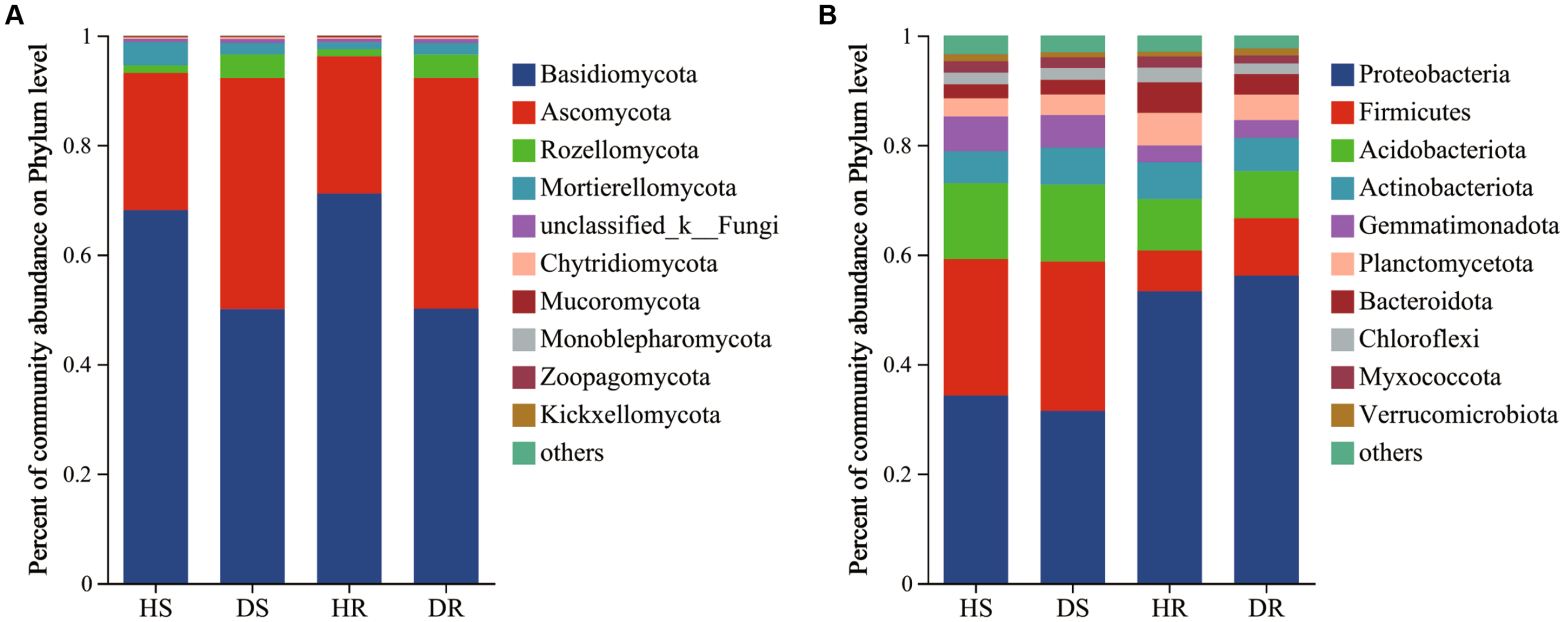
Microbial community composition of the root surface soil and rhizosphere soil of healthy and diseased strawberry at the phylum level, **(A)** fungal community composition; **(B)** Bacterial community composition.

In the fungal community, Basidiomycota (50.05%–71.13%) and Ascomycota (25.05%–42.17%) were dominant phyla across all soil samples. Additionally, the top 10 genera in terms of species abundance are listed (Supplementary Figure 3). Dominant genera included *Clitopilus* (32.20%–47.75%), *Solicoccozyma* (5.94%–16.42%), *unclassified_c_Agaricomycetes* (6.89%–13.72%), *Trichoderma* (2.95%–4.99%), and *Colletotrichum* (0%–6.01%), as shown in Figure 2A.

There were pronounced differences in fungal community structure between healthy and diseased plants. Conversely, fungal community structures in different ecological niches of strawberry soil exhibited similarity. In diseased strawberry plants, the relative abundances of Basidiomycota, *Clitopilus*, *unclassified_c_Agaricomycetes*, and *Pilidium* decreased, while the relative abundances of Ascomycota, Rozellomycota, *Trichoderma*, and *Colletotrichum* increased when compared with those in healthy plants. Notably, the relative abundances of the genus *Colletotrichum* in DR and DS were 6.96 and 6.01%, respectively, whereas it was less than 1% in HR and HS. The observation might be linked to the occurrence of strawberry root rot.

In the case of bacterial community (Figure 2B), Proteobacteria (31.43%–51.13%), Firmicutes (9.41%–27.31%), Acidobacteriota (8.59%–14.05%), Actinobacteriota (5.81%–6.74%), Planctomycetes (3.33%–5.94%), and Bacteroidota (2.54%–5.58%) were the dominant phyla across all soil samples. Additionally, the top 10 genera in terms of species abundance are listed (Supplementary Figure 3). Dominant genera included *Bacillus* (5.00%–20.80%), *Sphingomonas* (2.22%–5.64%), *uncultured_f_Gemmatimonadaceae* (1.92%–4.67%), and *Novosphingobium* (0.40%–5.35%) at the genus level.

In the comparison between diseased and healthy strawberry plants, there was a decrease in the relative abundance of Firmicutes in the root surface soil of diseased plants (Figure 2B). Although the species composition of most bacteria showed no significant differences between diseased and healthy plants, bacterial community structure exhibited significant differences among the different ecological niches within healthy or diseased plants, indicating that different ecological niches of strawberry roots have specific effects on bacterial taxa selection. In other words, bacterial community assembly in strawberry roots is primarily driven by organ-specific ecological niches. The occurrence of strawberry disease has minimal impact on root bacterial community structure.

### 3.4. Soil microbial co-occurrence networks

**Figure 3:**
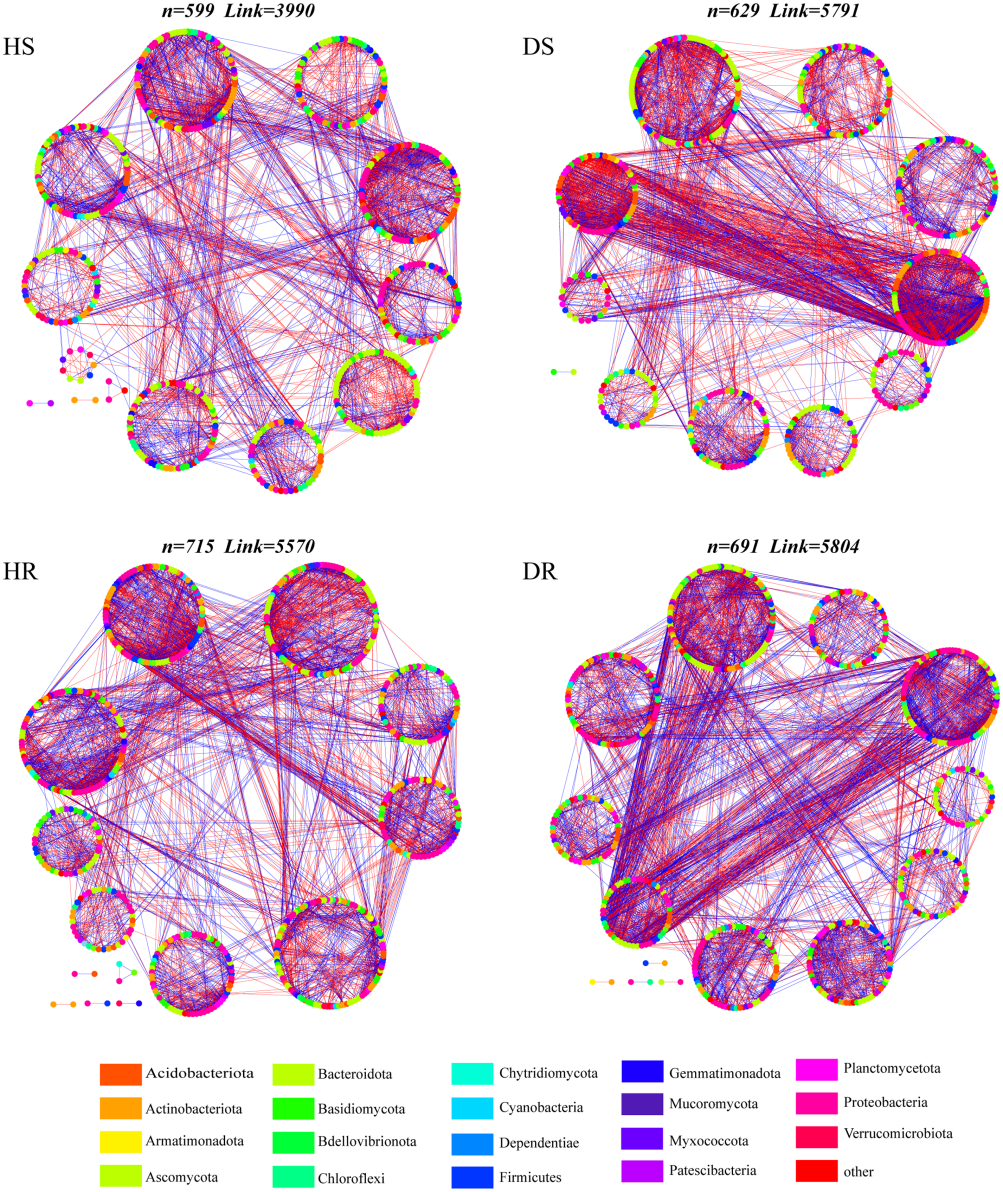
Visualizations of microbial co-occurrence networks in different soil samples. The nodes are colored according to bacterial phylum and fungal phylum. The circle surrounded by nodes represents the module. Edge color represents positive (green) and negative (red) correlations.

To delve into the interconnectivity within the root-associated microbial communities of healthy and diseased soils, a comprehensive co-occurrence network analysis was conducted. Within the networks, the fungal nodes predominantly stem from eight distinct phyla, among which Basidiomycota, Ascomycota, and Rozellomycota exhibit widespread distribution. With regard to bacteria, nodes predominantly hail from 11 diverse phyla, with Proteobacteria, Firmicutes, Acidobacteriota, and Actinobacteriota displaying high prevalence (Figure 3). Additionally, the topological properties of the microbial co-occurrence networks in the rhizosphere soil of healthy and diseased strawberry plants were computed separately (Supplementary Table 1). The microbial interaction network in the diseased plants had higher node and edge numbers than those in the healthy plants, indicating a more intricate network. However, the diseased plant network had lower modularity and higher average path length.

**Figure 4:**
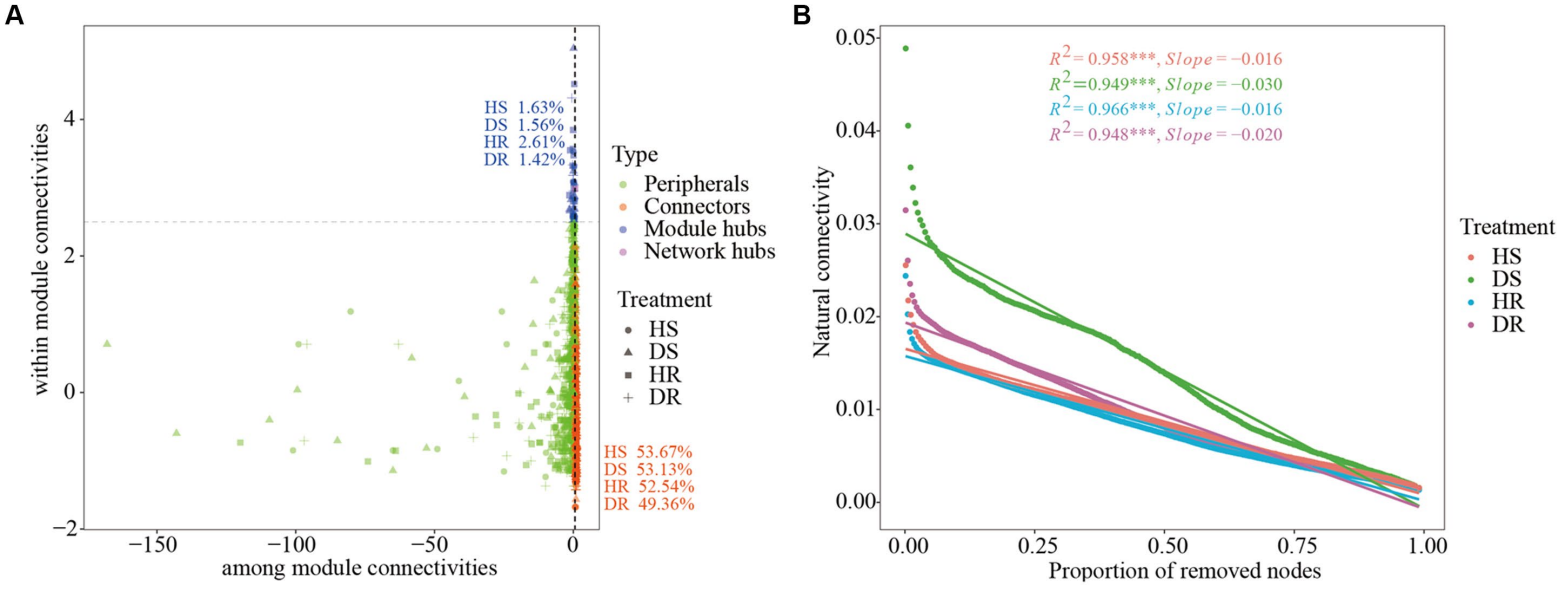
**(A)** Zi–Pi plot shows the distribution of OTUs based on their topological roles with different soil samples. **(B)** Changes in the natural connectivity of different soil samples.

The keystone species were evaluated by calculating the intra-module connectivity (Zi) and inter-module connectivity (Pi) of nodes in the network. The proportions of module hubs in the co-occurrence networks of HS and HR were 1.63% and 2.61%, respectively, which were higher than those in the co-occurrence network of DS and DR (1.56% and 1.42%, respectively). The proportions of connecting nodes (Connectors) of HS and HR were 53.67% and 52.54%, respectively, which were also higher than those of DS (53.13% and 49.36%, respectively; Figure 4A). The results show that the connectivity within and between modules of healthy plant soil co-occurrence network were higher.

The resistance of the microbial interaction network to plant infection and network structure stability were tested by removing nodes to alter the natural connectivity of the network. The results showed that by removing the same proportion of nodes, the natural connectivity of the microbial interaction network of the healthy plant had a gentler downward trend than the natural connectivity of the diseased plant (the slope of the fitting curve was lower; Figure 4B), indicating that the microbial interaction networks in HS and HR had higher network stability and higher resistance to adversity than those of DS and DR.

### 3.5. Soil microbial functional prediction

**Figure 5:**
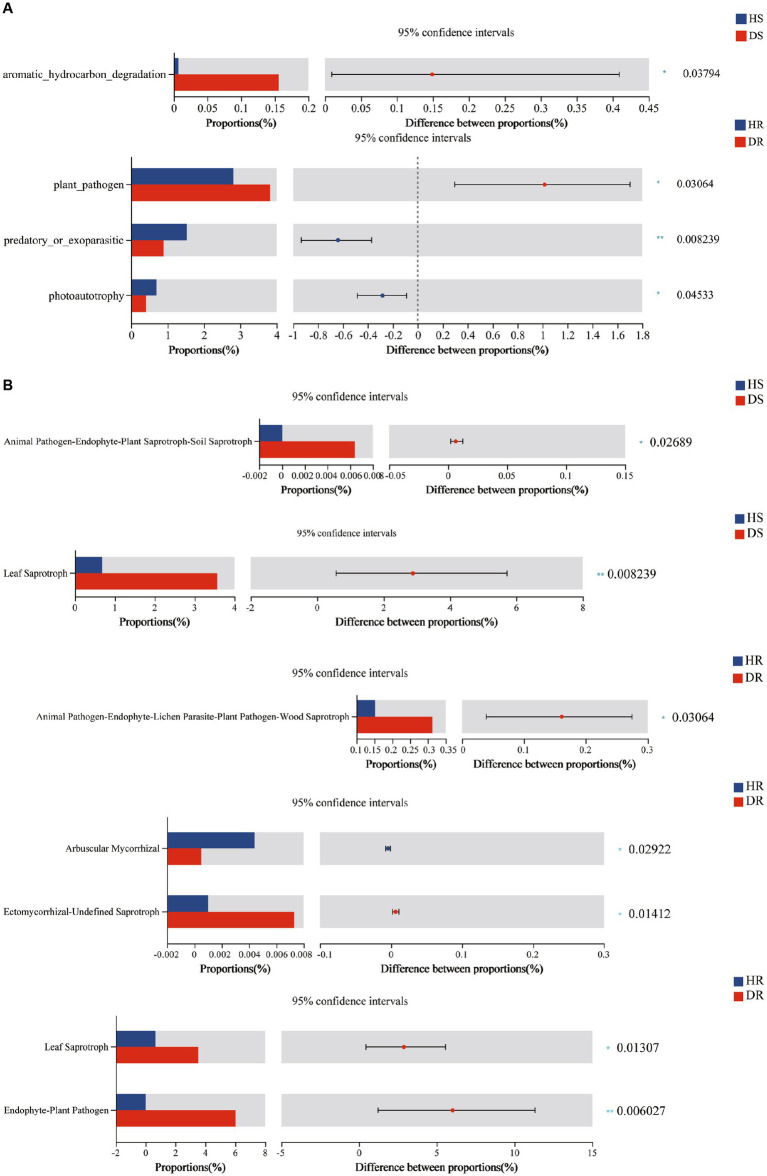
**(A)** Prediction results of root surface soil and rhizosphere soil FAPROTAX function of healthy and diseased strawberry plants, **(B)** Prediction results of root surface soil and rhizosphere soil FUNGuild function of healthy and diseased strawberry plants. The abscissa of the left histogram represents the average relative abundance of a feature in different groups. The ordinate represents the grouping category of different features or pairwise comparison in the task group. Different colors represent different groupings. The rightmost is *p*-value, ^*^0.01 < *p* < 0.05, ^**^0.001 < *p* < 0.01, ^***^p < 0.001.

By employing FAPROTAX, a comprehensive functional prediction was conducted on the bacterial communities within the root systems of both healthy and diseased strawberry plants. A total of 60 functional categories were anticipated across all soil samples, among which a plethora of bacterial functional types exhibited significant enrichment, including chemoheterotrophy, aerobic chemoheterotrophy, nitrogen fixation, ureolysis, nitrate reduction, plant pathogen, and chitinolysis. Analysis of functional classification discrepancies between the root-associated bacteria of healthy and diseased strawberry plants was performed using the Kruskal-Wallis rank-sum test, subsequently revealing distinctly altered bacterial functional assemblages in root surface soils (*p* < 0.05; refer to Figure 5A). The findings unveiled prominent differences in bacterial functional pathways across different soil samples. Notably, the relative abundance of aromatic hydrocarbon degradation bacteria was significantly higher in the root surface soil of diseased plants than in soils of healthy plants. Furthermore, in the rhizosphere soil of diseased plants, there was a significant reduction (*p* < 0.05) in the relative abundance of photoautotrophy and predatory or exoparasitic bacteria, while the relative abundance of the plant pathogen type displayed a notable increase when compared with that in healthy plants (*p* < 0.05).

Utilizing FUNGuild for functional prediction analysis of fungi within the rhizosphere and root surface soils of both healthy and diseased strawberry plants, a total of 17,574 OTUs were annotated into seven nutritional guilds, including Pathotroph, Symbiotroph, Pathotroph-saprotroph-Symbiotroph, Pathotroph-Saprotroph, Pathotroph-Symbiotroph, Soprotroph, and Soprotroph-Symbiotroph. Using the Kruskal-Wallis rank-sum test for prediction of functional subcategories of soil fungi, notable differences were observed between diseased and healthy plants at the root surface soil level (Figure 5B). Specifically, the Leaf Saprotroph guild in the root surface soil of diseased plants surpassed that of healthy plants at a highly significant level (*p* < 0.01). In the rhizosphere soil, the relative abundance of Leaf Saprotroph guild was also significantly higher in diseased plants. In addition, notably, the relative abundance of Endophyte-Plant Pathogen in diseased plants was significantly higher (*p* < 0.01), suggesting a direct correlation with the onset of strawberry plant disease.

### 3.6. Isolation and identification of cultivable soil microorganisms

**Figure 6:**
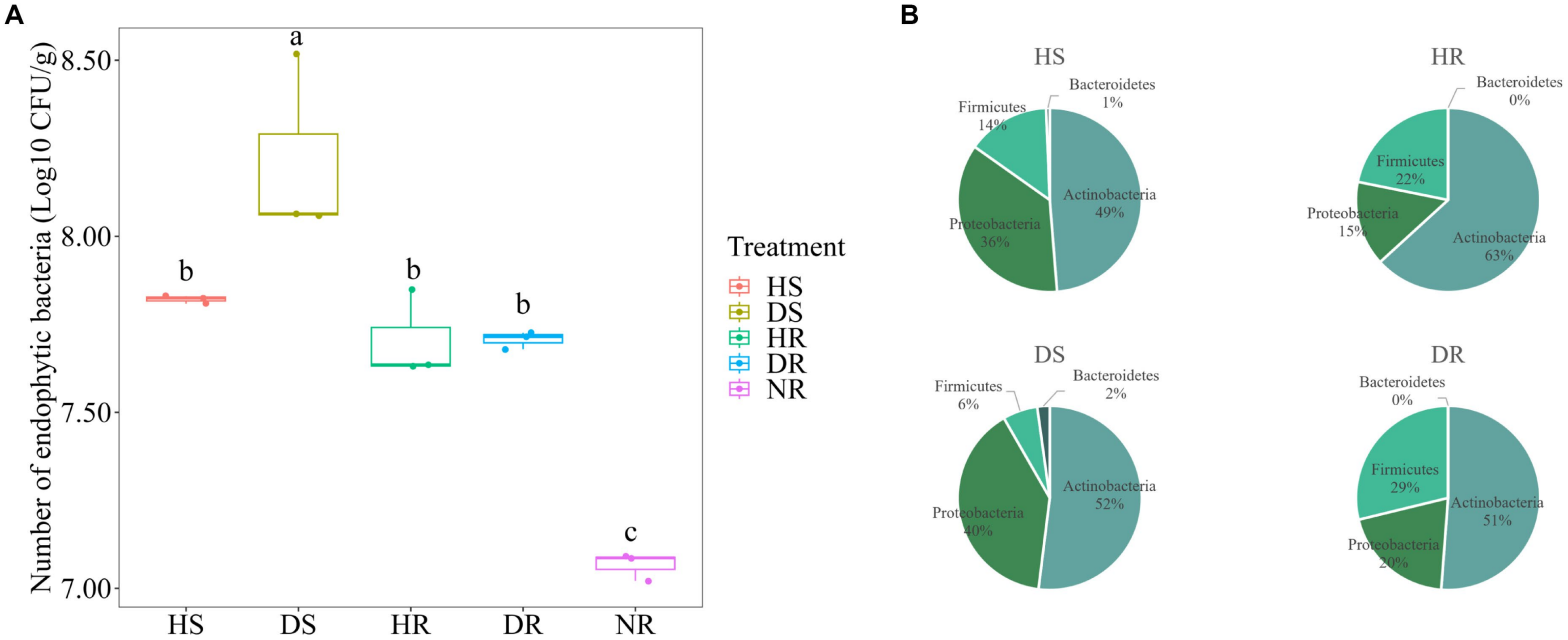
**(A)** Isolation frequency of the bacteria with different soil samples. **(B)** Community composition of culturable bacteria at phylum level in different soil samples.

The number of bacterial taxa in diseased plants was higher than that in in healthy plants. Additionally, the number of isolated bacteria in different ecological niches of strawberry roots had the following order: root surface > rhizosphere > non rhizosphere (*p* < 0.05). The quantity of bacteria in the root surface of diseased plants was significantly higher than that in healthy plants, as depicted in Figure 6A (*p* < 0.05). In the present study, further isolation and purification were carried out on the dilution plate-isolated bacteria, resulting in a total of 912 strains. After removing redundancy through analysis of 16S rDNA gene similarity, 192 bacterial species were identified. The 192 strains were distributed across four phyla, six classes, 20 orders, 41 families, and 75 genera.

Culturable bacteria in strawberry root surface soil belonged primarily to the phyla Actinobacteria, Bacteroidetes, Proteobacteria, and Firmicutes, with Actinobacteria being more prevalent in the HR (Figure 6B). Furthermore, species-level richness Venn diagram and species abundance pie chart were generated (Supplementary Figure 4). Among the microbial isolates from DR, the greatest diversity was observed in prokaryotic microorganisms. Notably, certain species, such as *Pseudomonas alcaligenes* (3%), *Pseudomonas aeruginosa* (2%), and *Chryseobacterium cucumeris* (2%), were isolated exclusively from the DR. *Luteimonas panaciterrae,* a ginseng-associated bacterium, was detected at a proportion of 4% in the root surface of healthy plants but was absent from other soil samples.

## 4. Discussion

Soil physicochemical characteristics are crucial indicators of soil quality. In the present study, there were no significant differences in most physical and chemical indexes between HR rand DR; however, there were significant differences in TN (total nitrogen), SOM (soil organic matter), OP (Olsen phosphorus), and AK (available potassium) between HR and DR, and NR. The variations in soil physicochemical properties across different root ecological niches were much more substantial than the differences between healthy and diseased plants. Notably, the pH values of both HR and DR were significantly higher than that of NR (*p* < 0.05), Soil pH can directly reflect soil acidity and alkalinity, and rhizosphere soil has a certain buffering capacity for pH changes. Roots can compensate for unbalanced cation-anion absorption at the soil-root interface by releasing H^+^ or OH ^−^, thereby significantly changing rhizosphere pH (Riley and Barber, 1969). Youssef and Chino (1989) found that the difference in rhizosphere and non-rhizosphere soil pH was more dependent on plant species and initial non-rhizosphere soil pH, rather than nitrogen source or nitrogen application level. Root-mediated pH changes involve many basic processes and a variety of environmental factors (Hinsinger et al., 2003). The interaction between soil pH and strawberry roots needs further study. A deficiency in soil elements such as N, P, and K increases plant disease risk significantly (Walters and Bingham, 2007). Significant differences in TN and AN (available nitrogen) were observed between HR and DR, which is consistent with the findings of Liao et al. (2022) in their study on root rot disease in Sichuan pepper plants, where they observed significant reductions in TN and AN levels in the rhizosphere soil of diseased plants when compared with healthy ones. The trends suggest correlation between soil TN and AN and the occurrence of strawberry plant disease. However, further experimental validation is required to definitively establish and explain the relationship.

The intricate interplay of root-associated microbial communities has a pivotal role in host plant immunogenic defense (Stringlis et al., 2018). Such communities not only suppress disease (Carrión et al., 2019) but also provide essential nourishment (Zhang et al., 2019) and safeguard against both biotic and abiotic stresses. Therefore, alterations in soil microbiota can serve as indicators of plant growth conditions and serve as a crucial metric for gauging soil health (Lehmann et al., 2020). High-throughput sequencing revealed notable discrepancies in fungal species composition between diseased and healthy strawberry plants. Conversely, within the diverse ecological niches of the strawberry root system, the variance in fungal species composition remained modest. Among diseased plants, the relative abundances of dominant phyla such as Basidiomycota and *Clitopilus* were markedly lower when compared with those in healthy plants. Conversely, the relative abundances of phyla such as Ascomycota, Rozellomycota, and genus *Trichoderma* were elevated significantly in diseased plants. This is similar to Yang et al. (2020) who found that the relative abundance of Ascomycota in strawberry plants increased by 20% after infection with powdery mildew, while the relative abundance of Basidiomycota decreased by 20%. The trends are consistent with the findings of Zheng et al. (2022) who reported reduced abundance of Basidiomycota in healthy tobacco roots when compared with that in tobacco afflicted by Rhizoctonia root rot. Moreover, the congruence with the findings of Gu et al. (2020) on tomato plants underscores the interconnectedness between decreased Basidiomycota and increased Ascomycota abundances in DR. The results indicate that the decrease and increase in relative abundance of Ascomycota and Basidiomycota were closely related to plant disease. However, whether the relationship between Basidiomycetes and Ascomycota is nutritional, attributable to niche competition, or antagonistic needs to be verified further. Notably, at the genus level, the relative abundance of *Clitopilus* decreased by 15.39% in DR, while the relative abundance of *Colletotrichum* increased by 6.01%, when compared with HR. The increased presence of *Colletotrichum* could potentially be a direct cause of strawberry disease. *Colletotrichum* spp., causing anthracnose, poses a devastating threat to various crops globally, and extensive research indicates that *Colletotrichum* is a major pathogen in strawberry fields. Anthracnose is likely to occur throughout the growth cycle of strawberries (Curry et al., 2002; Rahman and Louws, 2017). The increased *Colletotrichum* relative abundance could be a significant factor triggering strawberry disease. Bacterial species composition varies significantly across different ecological niches within the strawberry root system. However, the differences between diseased and healthy plants are minimal, with only Firmicutes exhibiting lower relative abundance in the rhizosphere and root surface soils of diseased plants when compared with those of healthy plants.

The alpha diversity of fungal communities in diseased plants surpassed that in healthy plants. Simultaneously, differences in alpha diversity indices among various ecological niches within the strawberry root system are less conspicuous. The beta diversity of root surface soil and rhizosphere soil from diseased plants clusters within a shared confidence interval, implying high fungal similarity within the diseased plant rhizosphere. Previous studies suggest a negative correlation between fungal quantity/diversity and soil health, as certain fungi can hamper plant growth by disrupting root systems, thereby impacting plant vitality (Yim et al., 2013; Emmett et al., 2014). Siegieda et al. (2023) found that the alpha diversity index of strawberry fungal community in non-rhizosphere and rhizosphere soil was higher than that of healthy plants. At the same time, Su et al. (2022) found that the rhizosphere soil microbial community diversity and richness of strawberry anthracnose plants were higher than those of healthy strawberry rhizosphere soil, which was consistent with the results of this study. Conversely, bacterial community alpha diversity follows a distinct trend when compared with fungal communities. No marked distinction is evident between the root surface soils of diseased and healthy plants. However, significant differences are observed in the bacterial alpha diversity indices across distinct ecological niches within the strawberry root system under similar treatment. There are few studies on the microorganisms of different niches of strawberry roots, and the research on different niches of other plant roots is referred to. The finding is consistent with Wang et al. (2023), who noted that changes in hydrodynamics and physicochemical properties can alter microbial diversity in aquatic environments significantly, but it does not change the alpha diversity of rhizosphere and endophytic bacteria significantly.

Research has indicated significant variations in bacterial communities across different ecological niches in tomatoes (Han et al., 2020). Gdanetz and Trail (2017) affirmed that wheat microbial community establishment is influenced predominantly by location-based ecological niches and host species, with minimal impact from soil, climate factors, or fertilization practices. The alpha diversity of bacteria in the rhizosphere of diseased plants was lower than that of healthy plants, but it did not reach a significant level, which was contrary to fungal diversity. In summary, in comparison to healthy plants, diseased plants display elevated fungal diversity and decreased = bacterial diversity. Furthermore, fungal communities exhibit consistent patterns in both species composition and diversity across different ecological niches within the root system. In contrast, bacterial communities display significant variations among the niches, demonstrating a gradient within the root environment, indicative of regional stability. This might reflect bacterial adaptation to long-term environmental change (Gibbons et al., 2014; Liu et al., 2018).

Using co-occurrence networks to depict the intricate interrelationships among microorganisms, we employed perturbation of natural network connectivity through random node removal to evaluate network structural stability. The results revealed a stability ranking for microbial interaction networks across the treatments as follows: HR > HS > DR > DS. The results show that the microbial interaction network has large difference in community stability between diseased plants and healthy plants, and there are differences in different niches of strawberry roots. Fungal community diversity of the diseased plants increased, and the microbial interaction network was more complex than that of the healthy plants; however, microbial interaction network stability was reduced and the degree of modularization was low. The higher the degree of network modularity, the greater the number of modules in the network. When the interactive network is subject to external interference, the existence of such modules can control the interference within one module and avoid its spread to other modules (Wang et al., 2016). Such findings underscore that the rhizosphere microbial community of healthy strawberry plants possesses a certain degree of structural and functional stability. However, mere diversity and richness are insufficient to ensure the stability of microbial community structure and function. Therefore, the microbial community in the rhizosphere of healthy plants exhibits greater resilience and stability when confronted with pathogenic interference.

Significant enrichment was in the Endophyte-Plant Pathogen, Leaf Saprotroph, and other Saprotroph guilds in diseased plants was observed following FUNGuild prediction analysis. The observations are likely associated with the decomposition of root tissues following plant infection. In addition, functional prediction analysis of bacterial communities within the root surface soils of healthy and diseased strawberry plants using PAFPROTAX revealed marked enrichment in aromatic hydrocarbon degradation and plant pathogenic functional categories in the diseased plants. Notably, previous studies have shown that *Pseudomonas* species possess aromatic compound-degradation capacity (Lunt and Evans, 1970; Ahmed and Focht, 1973). Additionally, earlier cultivation-based investigations have reported notable presence of *Pseudomonas* in the root surface soil of diseased plants, encompassing species such as *Pseudomonas putida*, *Pseudomonas alcaligenes*, and *Pseudomonas umsongensis*. *Pseudomonas putida*, in particular, has found extensive applications in industrial pollution mitigation (Henríquez et al., 2023) and serves as a biological control agent against pathogens (Ashajyothi et al., 2023). Furthermore, a recent study (Yi et al., 2022) revealed that benzo[a]pyrene (BaP) addition enriched microbes associated with aromatic compound degradation (Sphingomonas, Bacilli, Fusarium). Overall, the findings suggest that upon exposure to pathogens, strawberry plants might secrete specific root exudates, consequently fostering the enrichment of aromatic hydrocarbon-degrading bacteria. Conversely, in the rhizosphere of healthy plants, substantial enrichment of predatory or exoparasitic and photoautotrophic guilds was observed. Notably, following plant infection, root-associated bacterial communities shifted from autotrophic to saprotrophic guilds.

In the present study, bacterial groups were isolated and the number of microorganisms in the rhizosphere and root surface of both healthy and diseased plants was much greater than that in the non-rhizosphere. This is because the secretions produced by the root system can be used as a nutrient source by microbes for growth and reproduction (Bais et al., 2006). Consequently, due to root exudates, microbial abundance exhibited the following pattern: root surface microbes > rhizosphere microbes > non-rhizosphere microbes, suggesting a higher level of connection between plants and root surface microbes than with rhizosphere and non-rhizosphere microbes. Moreover, the quantity of root surface microbes in diseased plants exceeded that in healthy plants significantly. This might be linked to the degree of disease progression, as during the middle to later stages of disease, root rot releases organic substances that provide nutrients, resulting in substantial bacterial reproduction. Furthermore, there was a substantial increase in Actinobacteria in the rhizosphere of healthy plants. Considering soil Actinobacteria often produce antibiotics and promote plant growth (Zhao et al., 2023), they play a crucial role in generation of soil-borne antimicrobial substances. The decrease in the proportion of soil Actinobacteria can lead to weakened soil disease resistance and influence the regulation of soil microbial ecology (Doumbou et al., 2001). Further isolation and identification of bacteria showed that the relative abundance of *Pseudomonas alcaligenes*, *Pseudomonas aeruginosa*, and *Pseudomonas putida* in the root surface of the diseased plants was relatively high, and *Pseudomonas putida* could easily degrade and utilize aromatic compounds and other substances (Furuno et al., 2010). In addition, Li et al. (2019) observed that 1-aminocyclopropane-1-carboxylate (ACC) is a major component of plant rhizosphere secretions and can be chemotactic for *Pseudomonas putida*. Therefore, when strawberry is attacked by pathogens, some root exudates will be secreted to attract *Pseudomonas putida* to colonize roots, and the key substances need to be explored further. In such a context, various indicators suggest that saprotrophic bacteria increase substantially in the rhizosphere soil of diseased plants, while a decrease in Actinobacteria implies weakened soil disease resistance, enhanced pathogenicity, microbial community imbalance, and degraded soil microbial ecosystem.

## 5. Conclusion

In the present study, the relationship between strawberry root rot occurrence and rhizosphere microbial community structure was explored across different rhizosphere soil and root surface soil niches in healthy and diseased strawberry. According to the results, incidence of strawberry root rot influenced microbial community diversity and drove fungal community composition in strawberry roots. However, the different niches selected specific bacterial groups, while the incidence of strawberry root rot had minimal effect on the bacterial community structure of strawberry roots. After strawberry was infected with pathogens, microbial interaction network stability also decreased, and more endophytic-plant pathogen groups and saprophytic functional groups were enriched, which led imbalance in soil microbial community structure. In addition, the number of culturable bacteria in the root surface soil of diseased plants was significantly higher than that in healthy plants. In summary, the present study provides a comprehensive analysis of rhizosphere microecology and root microbial community composition in healthy and diseased strawberry plants, offering a theoretical basis for the prevention and control of strawberry root rot from a microbial ecology perspective.

## Data availability statement

The datasets presented in this study can be found in online repositories. The names of the repository/repositories and accession number(s) can be found at: the Picbio single reads for bacterial 16s rRNA gene and fungal ITS regions have been deposited in NCBI SRA database using accession code PRJNA1011218.

## Author contributions

MZ: Data curation, Formal analysis, Methodology, Writing – original draft. ZK: Data curation, Formal analysis, Methodology, Writing – original draft. HF: Formal analysis, Methodology, Writing – review & editing. XS: Methodology, Project administration, Writing – review & editing. QX: Methodology, Resources, Writing – review & editing. HL: Conceptualization, Writing – review & editing, Supervision. QG: Conceptualization, Writing – review & editing, Methodology, Visualization.

## Footnotes

^1^ https://blast.ncbi.nlm.nih.gov/Blast.cgi

^2^ https://cloud.majorbio.com

^3^ http://www.arb-silva.de

^4^ http://www.mothur.org/wiki/Calculators

^5^ https://github.com/wqssf102/fastnc
