# Supplementary material for: Rhizosphere microbial ecological characteristics of strawberry root rot
Source: Front Microbiol. 2023 Nov 16;14:1286740. doi: 10.3389/fmicb.2023.1286740 (PMC10687216; doi:10.3389/fmicb.2023.1286740)
Supplement: Supplementary file 1 [file Data_Sheet_1.docx]

Supplementary Material

Rhizosphere Microbial Ecological Characteristics of Strawberry Root Rot

Meilin Zhang, Zirong Kong, Huijing Fu, XiaoLong Shu, Quanhong Xun, Hangxian Lai

*** Correspondence:** Qiao Guo
shuiyiwei83@163.com

# Supplementary Figures and Tables


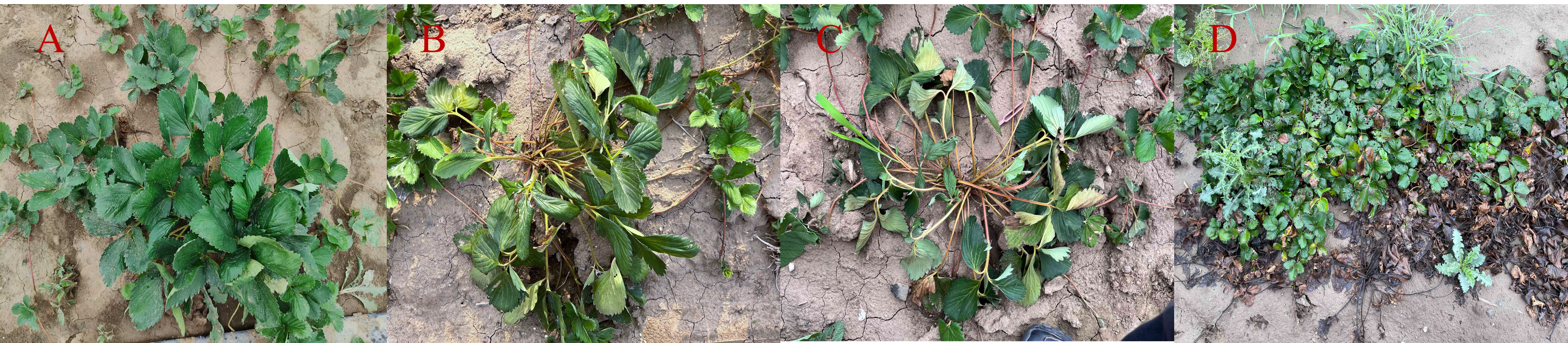


Figure 1. The incidence of strawberry root rot in the field

Note: (A) healthy strawberry plants, (B-D) pathogenesis of strawberry root rot in the field.


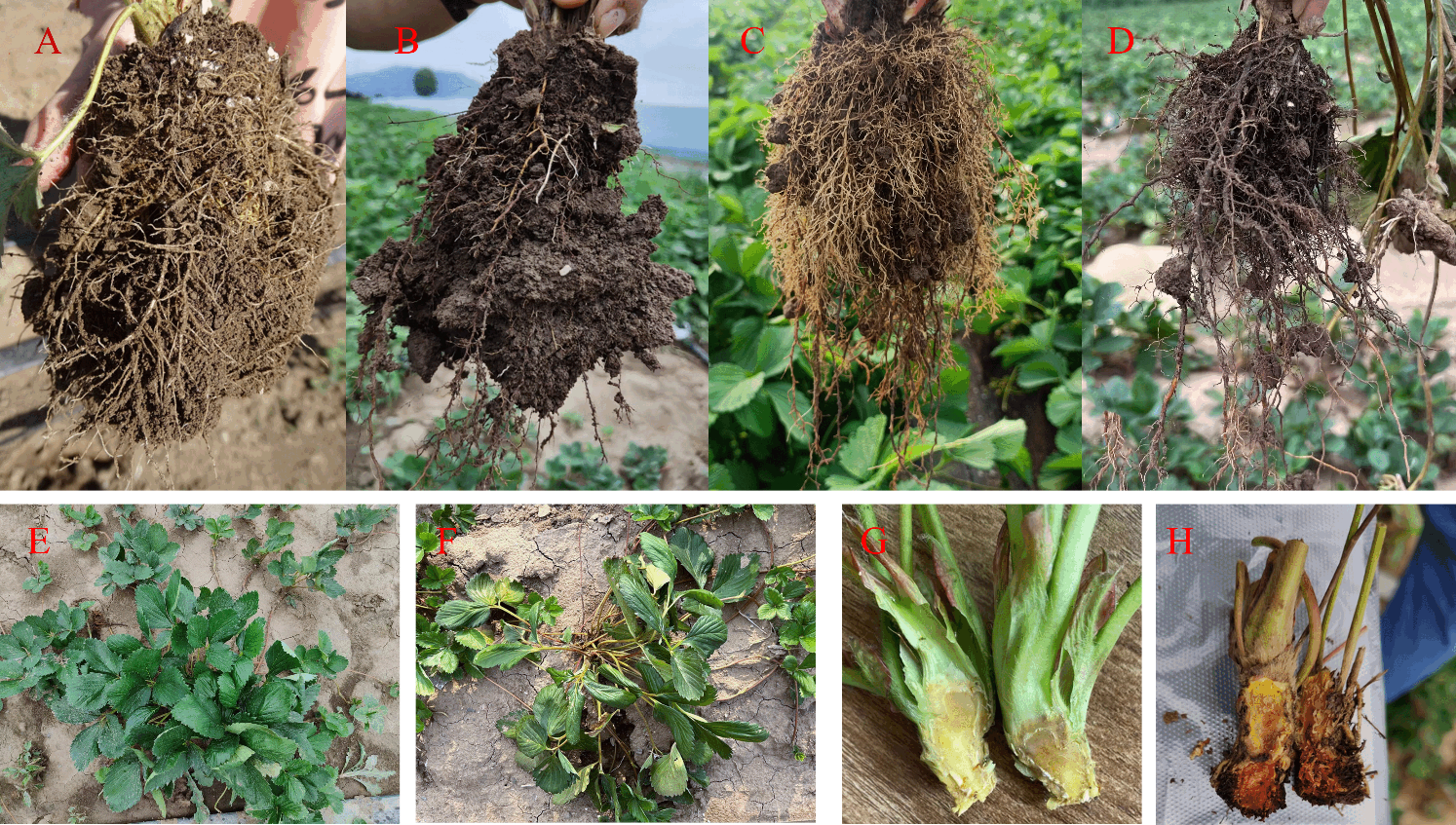


Figure 2. Field incidence of strawberry root rot. Red middle plant and root anatomy of diseased and healthy plants

Note: (A C E G) healthy strawberry plants, (B D F H) Strawberry root rot disease plants.


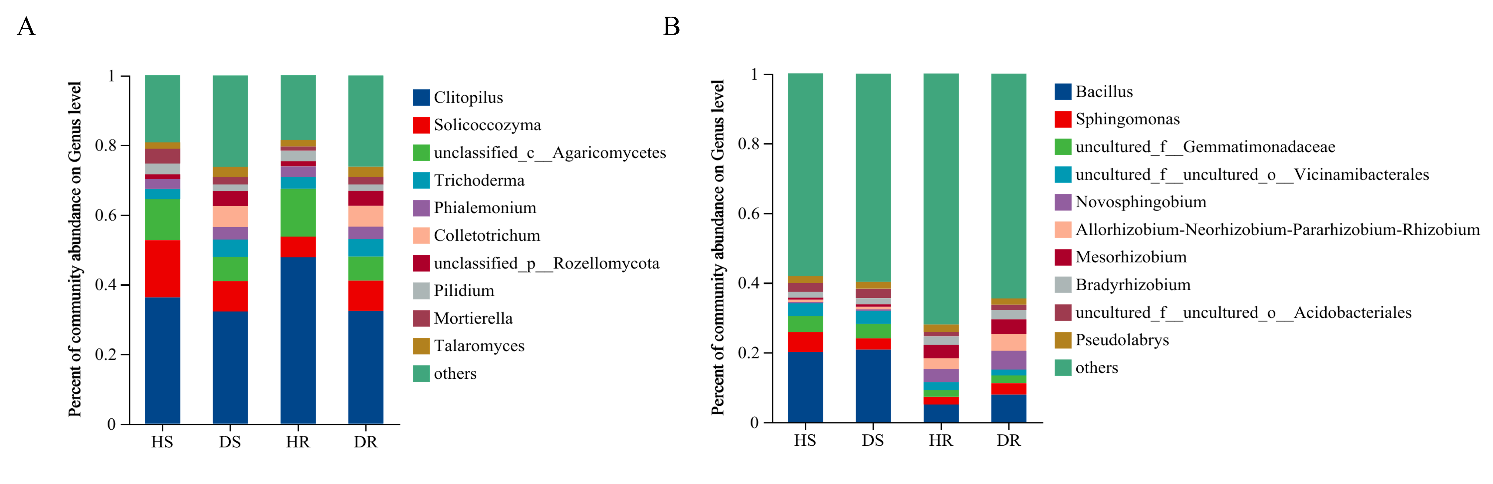


Figure 3. Microbial community composition of the root surface soil and rhizosphere soil of healthy and diseased strawberry at the genus level; (A) fungal community composition, (B) bacterial community composition


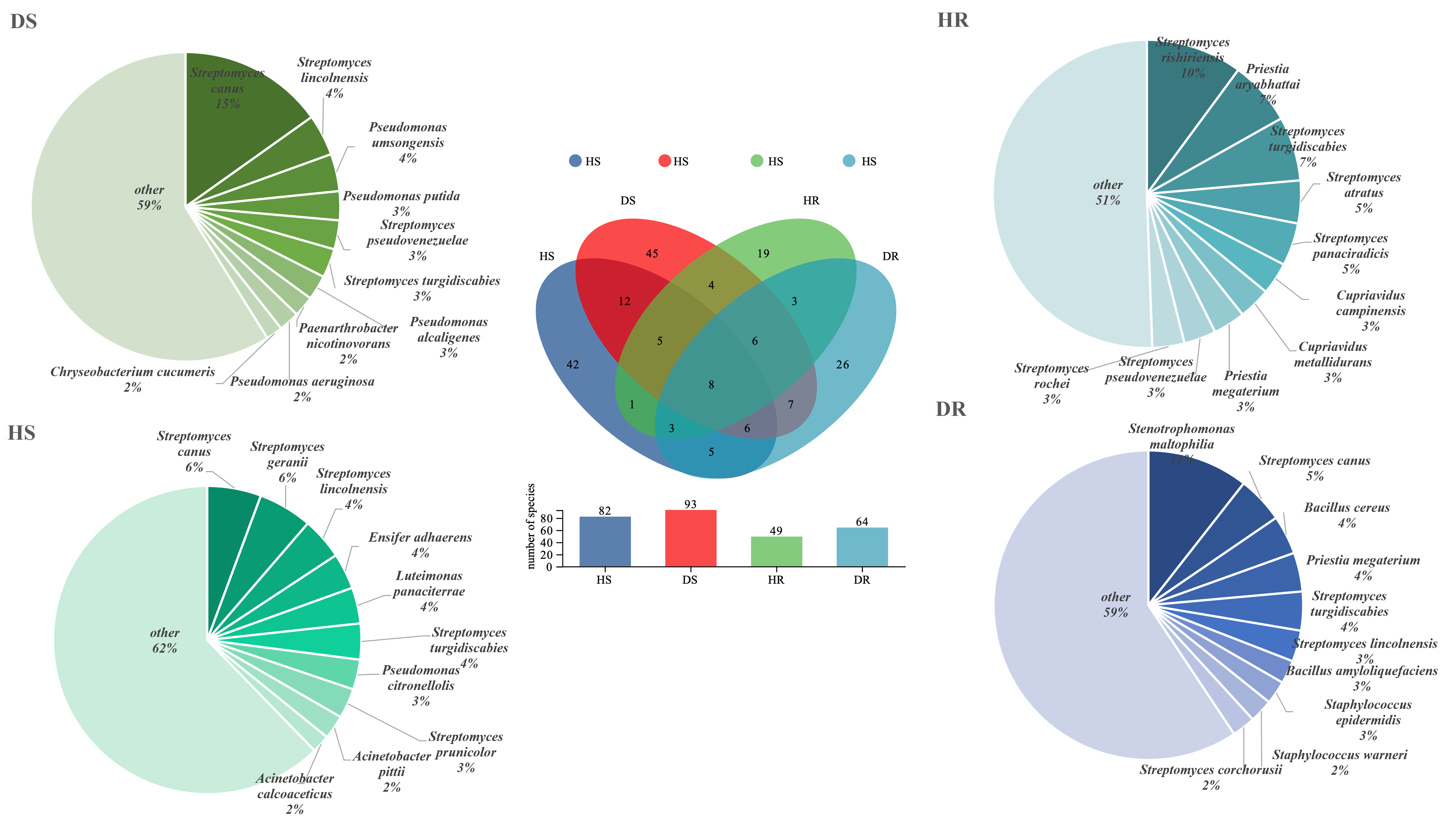


Figure 4. Number of bacterial species in different soil samples (venn) and the relative abundance of species (top 10) at the species level in different soil samples (pie chart).

Table 1. Co-occurrence network properties for microbial communities between the root surface soil and rhizosphere soil in healthy and diseased strawberry

| Treatments | Average degree | Diameter | Modularity | Average clustering coefficient | Eigenvector centrality | Average path length | Positivity (%) | Negative  (%) |
| --- | --- | --- | --- | --- | --- | --- | --- | --- |
| HS | 13.322 | 9 | 0.712 | 0.506 | 0.143244 | 4.543 | 58.67 | 41.33 |
| DS | 18.413 | 10 | 0.659 | 0.548 | 0.058318 | 4.414 | 61.35 | 38.65 |
| HR | 15.58 | 9 | 0.731 | 0.523 | 0.148055 | 4.425 | 53.72 | 46.28 |
| DR | 16.799 | 9 | 0.672 | 0.517 | 0.103768 | 4.385 | 52.03 | 47.79 |
